# Metaheuristic approaches in biopharmaceutical process development data analysis

**DOI:** 10.1007/s00449-019-02147-0

**Published:** 2019-05-22

**Authors:** Nishanthi Gangadharan, Richard Turner, Ray Field, Stephen G. Oliver, Nigel Slater, Duygu Dikicioglu

**Affiliations:** 1 Department of Chemical Engineering and Biotechnology, University of Cambridge, Cambridge, UK; 2 Cell Sciences, Biopharmaceutical Development, Medimmune, Cambridge, UK; 3 Department of Biochemistry, University of Cambridge, Cambridge, UK

**Keywords:** Biopharmaceutical process development data, Missing data handling, Interactive data visualisation, Meta-feature selection, Data modelling

## Abstract

There is a growing interest in mining and handling of big data, which has been rapidly accumulating in the repositories of bioprocess industries. Biopharmaceutical industries are no exception; the implementation of advanced process control strategies based on multivariate monitoring techniques in biopharmaceutical production gave rise to the generation of large amounts of data. Real-time measurements of critical quality and performance attributes collected during production can be highly useful to understand and model biopharmaceutical processes. Data mining can facilitate the extraction of meaningful relationships pertaining to these bioprocesses, and predict the performance of future cultures. This review evaluates the suitability of various metaheuristic methods available for data pre-processing, which would involve the handling of missing data, the visualisation of the data, and dimension reduction; and for data processing, which would focus on modelling of the data and the optimisation of these models in the context of biopharmaceutical process development. The advantages and the associated challenges of employing different methodologies in pre-processing and processing of the data are discussed. In light of these evaluations, a summary guideline is proposed for handling and analysis of the data generated in biopharmaceutical process development.

## Introduction

One of the major challenges in biopharmaceutical production is that unlike small molecule generic products it is impossible to manufacture identical copies of biologic products despite meticulously following well-defined analytical characterization and manufacturing techniques [1]. This raises a need for continuous real-time quality control and assurance in biopharmaceutical manufacturing, which emphasises the significance of enforcing process analytical technology (PAT) measures in a manufacturing process. Effective PAT implementation involves the use of complex process control strategies based on multivariate monitoring techniques. Software systems for multivariate data analysis enhance control and ensure overall process and product quality by detecting process deviations and identifying their root causes [2]. These high-throughput techniques generate large amounts of data containing real-time measurements of critical quality and performance attributes of the process, and if processed efficiently, can provide process improvement opportunities. Occasionally large amount of data are also generated as the result of an adaptation to superior cell culturing methods for improved production characteristics, such as the emergence and adoption of the perfusion mode of culturing [3].

Retrospective investigation of historical data comprised of batches of cultures using multivariate modelling techniques such as principal component analysis and partial least squares can help predict and control manufacturing processes towards achieving target productivity and quality end points [4]. The main objective of these retrospective analyses is to reveal insightful information from massive data sets to improve process understanding. These data can be used for the purpose of forecasting, identification of trends, relationships, pattern, and anomaly detection [5]. In line with recent initiatives and growing interest on handling of big data, biopharmaceutical and biologics industries launched similar programmes to mine and exploit their data further. Such an in-depth investigation would necessitate understanding the nature of these datasets and selecting suitable methods of analysis to extract the most relevant information out of these data.

Cell culture data pertaining to biopharmaceutical process development (BPD) deposited in databases are of multivariate time series type, and they possess temporal patterns. Time series analysis is a method of analysing longitudinal data, which allows the monitoring of a pattern of change over time rather than at a single discrete point in time [6]. The adoption of conventional statistical methods that consider time series observations as independent and uniformly distributed may harm prediction accuracy, as differences between time series values of adjacent examples are quite small and are strongly correlated [7]. To conduct an analysis on time series datasets, it is important to ensure that the time series is complete, so that auto-covariance and auto-correlation can be computed as a preliminary step to data mining [8].

Data mining techniques are broadly classified into supervised and unsupervised methods. Classification, regression and deviation detection fall under supervised techniques, where there is a prior knowledge of what the output values for the samples should be. These techniques essentially attempt to approximate the relationship between observable input data, such as those given by the process attributes and the output data, such as those given by the product attributes, in the best possible way. Pattern discovery, association rule discovery and clustering are, on the other hand, unsupervised techniques, which aim to infer the inherent information from unlabelled data points [5]. Each of these analyses consists of several steps. Pre-processing is a step that involves the identification of the problems in the dataset, such as the presence of gaps, and the selection of a suitable mechanism to handle the problem [9].

This review aims to investigate the stages involved in bioprocess data handling, and is structured as follows. The ‘Data Pre-processing’ section discusses different aspects of the data generated during process development, and the specific problems that impose challenges for their analysis, such as the incompleteness of the datasets, and presents possible strategies to handle these problems. This section also discusses the adoption of other key data pre-processing techniques for visualisation, selection of important features embedded in the data, and dimensionality reduction. The ‘Data Processing’ section provides an overview of the different methods available for model building, validation and optimisation. The review concludes by providing a critical evaluation of the adaptability of these approaches for both data pre-processing and processing in addressing the intrinsic properties of the data generated during BPD, and proposes a summary guideline for handling such datasets.

## Data pre-processing: handling of the missing information, visualisation of patterns, meta-feature extraction, and dimensionality reduction

### Handling of the missing information

#### Missing data in databases

One of the major challenges when dealing with historic cell culture data is the presence of missing values or gaps in the BPD databases. There are several reasons for the accumulation of these gaps, most of which are unavoidable. Missing information in the databases could be attributed to, but are not limited to sensor breakdown during operation, inconsistent sampling rates due to the nature of the process or to human intervention in sampling times, merging data acquired from different systems, or recording of measurements that fall outside of the range of the sensors. Furthermore, malfunctions in the data acquisition systems, any potential network outages, power black outs, data being logged in an incorrect format, human or machine errors in data recording, glitches in the data management software, and samples being flagged as having poor quality and subsequently being dropped from storage all contribute to the accumulation of missing information in databases [10, 11].

On many occasions, gaps arise due to the characteristics inherent to the production process itself. For instance, parameters such as the product concentration can be measured less frequently during early days of production than during late stages to reduce the amount of sample drawn out on early days of culture. However, a simple preventive action such as one that would exclude only three daily measurements in a bioprocess that is monitored for 2 weeks would mean missing information on 20% of the data in all available recordings for that parameter in the database. Similarly, a single missing value in one parameter can create a domino effect by also affecting a dependent parameter. For example, a missing recording in the elapsed generation number parameter can create consecutive gaps in the same parameter until the end of the bioprocess from that measurement forward, as well as creating gaps in another parameter such as the cumulative population doubling level, whose value is determined from the elapsed generation number.

The different types of activities, which cause the introduction of missing information into datasets, essentially provide the means to categorise nature of the missing data. Missing data are classified based on either the pattern of missing information or the missing data mechanism, i.e. the relationship between the observed parameter and the missing data. The patterns of missing information fall into a wide variety of structures such as that of (1) the univariate pattern, i.e. the gaps are only present in one parameter; (2) the unit non-response pattern, i.e. there is no data recorded for the duration of a single experiment out of a batch; (3) the monotone pattern, i.e. data are missing from a specific point forward due to a change in sampling regime or instrument leading to the omission of certain measurements; (4) the general pattern, i.e. missing data can be predicted from the observed values using a linear model; (5) the planned missing pattern, i.e. a measurement is intentionally omitted due to experimental design; or (6) the latent variable pattern, i.e. a missing data structure that explains the unavoidable differences in the imputed and the actual values of the missing data point. The relationship between the observed parameter and the missing data can be described as (1) missing completely at random (MCAR), i.e. the missing data does not depend on the measured data or the missing data itself; (2) missing at random (MAR), i.e. the missing data can be inferred from the measured data, but are not dependent on the missing data; or (3) missing not at random (MNAR); i.e. the missing data are not stochastic, but they depend on the missing data themselves [12].

It is important to consider seasonality, i.e. the recurring patterns, correlation among samples and smooth variations between examples while treating missing values in a time series data set [8]. The unavailability of even a limited fraction of data could yield to biased estimates of the existing effects, and thus overestimate the precision [13]. In any time series, missing information in more than 20% of the original dataset would potentially risk deviating away from the complete data pattern [6]. Thus, the handling of missing data becomes an essential step in data pre-processing prior to performing any further analysis. However, temporal patterns such as trends, seasonal, cyclic or irregular variations were reported to render handling of missing data in time series challenging [12].

#### Handling of the missing data

**Fig. 1:**
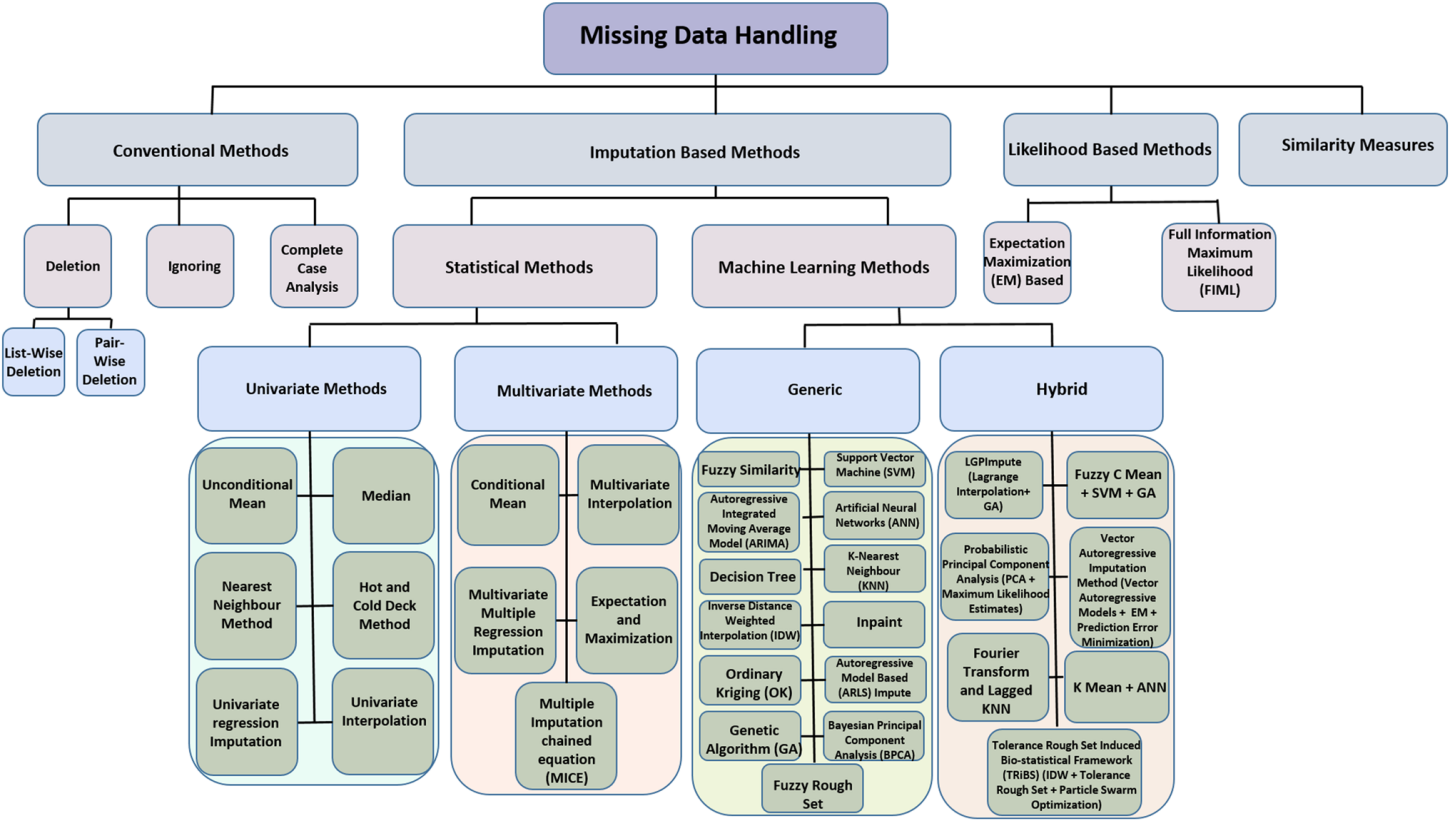
Different methods for handling missing data. Hierarchical tree depicting the classification of missing data handling methods, with each box representing a specific gap-filling method. The boxes are colour coded according to their hierarchy. Larger rectangular boxes in the same level of hierarchy represent members of the same cluster. The distances in the tree are not scaled to size or do not present mathematical significance, but only are a graphical representation (colour figure online)

Over the course of time, a multitude of approaches was developed to estimate the missing values in a dataset. These approaches can be broadly classified into four categories (Fig. 1): (1) conventional methods including complete case analysis, ignoring and deletion of data with missing components [6, 14], (2) imputation-based methods including statistical methods and machine-learning methods [12], (3) likelihood-based methods including expectation–maximization algorithms [15] and full information maximum likelihood [12], and (4) similarity measures [9]. Conventional methods are popular due to their simplicity, but are only effective if a low fraction of the complete dataset is missing. Adoption of these simple strategies could potentially induce bias in the resulting data analysis that follows this pre-processing protocol in the event that gaps comprise a high fraction of the dataset [9]. These methods were recently employed to handle the missing data from a BPD dataset, where less than 20% of the data were missing, and the methods were reported to perform efficiently without introducing bias into the downstream data analysis [16]. The likelihood-based approaches are present on the other end of the complexity scale. Although these approaches are flexible, they do not require ad hoc methods, and yield a satisfactory estimate of variance, they were shown to be computationally intensive in study on imputation of missing time series data on air pollutants due to the necessity to solve highly complex likelihood equations [13]. Similarity measures are yet another method for handling missing data, which results in a complete dataset without performing any imputation by simply replacing the missing values by best matched values between two time series using a dynamic time warping-like algorithm. Such a method was successfully used to impute missing data in EEG traces measured from a patient, which consisted of 3600 points of electric potential recordings [17]. Imputation has been increasingly attracting attention as an alternative strategy for handling of the missing data. Among different methods of imputation, machine-learning methods were reported to outperform statistical methods, providing significant improvement in prediction accuracy and reduction in error rates [5, 12]. Hybrid machine-learning methods present further superiority by combining the computational excellence of two or more methods. For instance, Resende et al. employed a combination of Lagrange interpolation with genetic algorithm on a simulated dataset to demonstrate improved imputation power by their proposed method [8]. Aydilek and Arslan used fuzzy C mean clustering in combination with Support Vector Machines and Genetic Algorithm on six standard machine-learning datasets to evaluate the efficacy of their hybrid algorithm [18].

#### Summary for handling of the gaps

Although a multitude of methods is available for imputation as discussed in the last subsection, few key aspects of the BPD datasets still render the task of gap-filling challenging. Conventional methods summarised here cannot be applied in a plug-and-play format to address the problem. Complexities embedded within the BPD dataset such as some parameters always being correlated across time, a single missing instance of a parameter creating consecutive missing values in the dataset, can all add to the challenge. Adherence to a standard production protocol can lead to multiple type of missing behaviour within a single parameter. For instance, BPD data have a missing mechanism known as MAR. This, when combined with, for instance, a planned missing pattern, can create a complex combination of missing behaviour, which renders it difficult to address using singular methods [14]. Moreover, studies reported that the best predictive accuracy did not necessarily lead to the lowest classification bias. This suggests that the modelling task, i.e. the following data-processing step, is imperative in the evaluation of the performance of any imputation method adopted for handling the missing data [19].

### Interactive data visualisation

Following the gap-filling protocol, the next task frequently adopted by data scientists aims to explore the patterns in time series data comprehensively [20]. A preliminary visual analysis is usually very convenient at this stage. Various visual analytics systems incorporating time-based visualisation, which serve to supplement temporal patterns, can be used for identifying patterns in the data. Several software tools with advanced visualisation capabilities are available for this purpose. Some of these tools generate only basic graphical representations (Leadline [21], Matisse [22], Visual BackChannel [23]), and others support enhancements that allow interactive visual queries to be made (ChronoLens [24]). More advanced tools execute clustering (SpiralView [25]), highlight recurring patterns (SpiralView), and some have multi-focus zooming techniques (SignalLens [26], RiverLens [27]). More elaborate toolboxes can be used to visualise an overview of statistical summaries of time series data (TimeBench Library [28]), and provide simultaneous views and pattern-matching capabilities (TimeSearcher 2 [29]). The advantage of using visual analytics approach is that it reduces knowledge discovery timelines and provides a deeper understanding of the process by allowing patterns in the behaviour of different parameters become comparable. Falcon is one such interactive data visualisation tool with an embedded feature that allows comparative visual analysis of long time series data that are sampled irregularly with sub-second precision. These patterns extracted from visual analysis can be further detailed out and be used for the formulation of hypotheses in complex time series data sets [20].

### Meta-feature extraction

Data collected from any standard bioprocess development protocol will comprise of some parameters, which are monitored or measured likely at regular intervals in time through interfering with the process, for instance, by sampling. These are generally referred to as offline parameters. Online parameters, on the other hand, provide continuous readings from automated control and data logging systems. There would be several orders of magnitude of difference between the frequency of the recordings collected through offline and online systems in any given period. Handling of the two different types of data together necessitates the online data to be pre-processed and made compatible for an integrated analysis along with the offline parameters. Conventional methods such as the computation of the mean, the standard deviation, the minimum and the maximum values across the dataset, and employing those values for the reduction of time series data were found to be unsatisfactory due to poor accuracy [6]. Using meta-feature selection algorithms is a complex approach to extract features from multivariate time series data, which would yield much higher accuracy than the basic methods would [30]. TClass is an algorithm based on the extraction of meta-features that model recurring substructures in the instances, such as strokes in handwriting recognition or local maxima in time series data [31]. The type of substructure is defined by the user, but are extracted automatically and used to construct attributes. The parameter space defined by meta-features is segmented into regions beneficial for classification using heuristic techniques [32]. Other algorithms available for meta-feature selection are Mutual Information [33], Document Frequency [34], Information Gain [35], and CHI algorithm [36, 37].

### Dimensionality reduction

It is desirable to reduce the dimensionality of the covariate space comprised of the multiple parameters monitored in the dataset to simplify modelling and analysis. This task aims to identify the critical parameters, which can explain the variance in the data efficiently, without necessitating the use of all measured parameters. Several methods of dimensionality reduction including but not limited to principal component extraction, partial least squares, and canonical correlations have been used for the analysis of time series data [34]. However, these methods do to account for the temporal aspect of time series datasets.

Template-matching framework is a time series data compatible system that can reduce the storage and computational requirement for classification problems. Niennattrakul et al. [38] proposed a shape-based template matching framework (STMF) for time series, which uses a shape-based averaging algorithm that employed a dynamic time warping distance space. Dynamic time warping distance was reported to be one of the most accurate distance measures for time series classification [38] as opposed to Euclidean distance, and other methods [39]. Unlike other data types, for which amplitude averaging approach could be used, time series data require a shape-based averaging approach since correlations exist among adjacent dimensions of the time series. The outputs from visual analytics software tools would help to isolate pairs of parameters that have similar behavioural patterns, i.e. similar templates. This would correspond to those parameters, which have similar influence on product concentration or any other output of interest in the case of cell culture data. These pairs of sequences could then be averaged using STMF to obtain one sequence capturing the essence of both parameters, thus reducing the number of parameters that had to be used for data analysis.

Feature selection, could be used as an alternative method for dimensionality reduction. It entails selecting a single feature or an optimally predictive feature subset of minimal size to achieve a target outcome [40]. Selection of suitable features may improve accuracy and efficiency of classifier methods [41]. In a high-dimensional time series data set where the relationship between the parameters is not known, the challenge is to extract the most relevant predictors that will contribute to successful models [42].

Time series data contain temporal ordering, which makes its feature selection different from that of static data. The target value of the former relates to the value of features in the previous time stamps as well as in the current time stamp. Therefore, feature selection in multivariate time series is a two-dimensional problem with two tasks: identifying the features and finding the window sizes of the features [43].

## Data processing: understanding the underlying relationship between input parameters and output measures

Models need to be constructed to understand and evaluate the interactions between different parameters that change over time and how they affect the outcome. These models can provide us with a clear understanding of the impact of each parameter on the process, and of how variations in each input parameter can influence the outcome. They can also be used as predictive tools to project on the future performance of ongoing cultivations as well as to hypothesise on the performance of novel process conditions.

### Relative contribution of input parameters on the outcome

Charaniya et al., developed a data analysis approach, which makes use of similarity scores between runs to estimate parameter weights [44]. This method assigns a weight to each parameter by comparing the similarity of that parameter profile between any two runs with the difference in the outcomes of these two runs. The weight of individual parameters is thus calculated, and this could be followed by dimensionality reduction, if necessary.

### Data modelling approaches

Following the pre-processing of the data, it could then be used to understand and model the interactions between the time series parameters. Conventional predictive modelling methods such as Hidden Markov Models, Recurrent Artificial Neural Networks and Dynamic Time Warping were commonly used to build predictive models for classification purposes [45–47]. However, these methods were shown to prove impractical for handling large datasets [30]. Hidden Markov Models operate with a large number of parameters that are set and monitored. Furthermore, they require major assumptions need to be made on concepts that are not readily available in real-world scientific datasets. These problems render their use impractical in the analysis of BPD. Recurrent artificial neural networks also have similar problems and require the user to experiment with and select the most suitable setting for many parameters, as well as to decide on a suitable network architecture [30]. In a comparative performance evaluation study, artificial neural networks were shown to converge to local minima and face overfitting problem, while support vector machines could find optimal global solutions and were reported to demonstrate excellent predictive accuracy and generalisation capability [48]. Partial Least Square Regression algorithm was designed specifically to operate with high-dimensional data. Partial Least Square Regression models combine dimensionality reduction and prediction using a latent variable model. In high-dimensional sample-limited problems where, for instance, a high number of parameters were monitored for each experiment but only a few samples were collected throughout the duration of each experiment, performing parameter selection avoids overfitting, and also provides accurate predictors for the model and yields more interpretable estimates with improved prediction capability [49]. Modified Partial Least Square Regression was proposed as a computationally simple and reliable method, which was able to overcome some of the drawbacks associated with Partial Least Square Regression and Principal Component Regression on BPD datasets. An example of such a problem is multicollinearity, i.e. the ability of any predictor variable in the model to be linearly predicted from the others with a substantial degree of accuracy, thus creating issues in modelling. Furthermore, Modified Partial Least Square Regression models were shown to be capable of providing a clearer interpretation of the correlation model than its predecessors [50].

Assumptions made in the construction of the models, as well as the method employed during model construction will introduce artefacts. For instance, a machine-learning model will intrinsically embody artefacts created by the training process. Once a model is built, a validation of the constructed model needs to be carried out to verify that it performs as expected, in line with their design objectives. Cross validation is a technique to assess how the results of a statistical analysis will generalize to an independent data set, and therefore becomes relevant in the evaluation of the performance of statistical models. Le et al. proposed a tenfold cross-validation scheme for training and evaluation of models [51]. The method also incorporated a model optimization step for each round of the tenfold cross-validation. It would be possible to employ a similar schema for assessing the predictive performance models generated by Support Vector Machines and Modified Partial Least Square Regression.

Evaluating the performance of the constructed model is crucial in identifying the best fitting model, which could then be used for future predictions. Pearson’s correlation coefficient and root mean square errors between the predicted and the actual outcome, which could be the product concentration or any one of the product quality attributes, could be potential indicators of the performance of the constructed model.

## Data mining for biopharmaceutical product development

There is a huge range of data pre-processing and processing methods available for addressing data mining problems. All these methods have their unique strengths and weaknesses. Some of these methods, which could be of potential interest in the analysis and modelling of bioprocesses, were discussed in the preceding sections. The selection of data pre-processing and processing methods, which are suitable to meet the needs and demands of datasets of different nature, necessitates a careful consideration. Hands-on experience with handling of the BPD data highlighted some unique challenges that require attention in handling of these datasets. These challenges arise specifically during the data pre-processing stage. It can be stressed that once the intrinsic irregularities or heterogeneities in the datasets are overcome, the dataset, regardless of its source and nature, becomes compatible with the standard data analysis and modelling tools available.

BPD data, which are generally multivariate time series, are also usually rank deficient and under-determined. This would mean that the data for each specific cultivation would have more parameters monitored than the number of data points available for these parameters. Typically, 20–25 different parameters could be monitored in a standard process with daily recorded readings available for 14–19 days of cultivation. Rank deficiency is an important challenge that makes a variety of pre-processing steps such as multiple imputation or artificial neural networks for gap filling incompatible for use with the dataset [52]. The main reason for this is that many algorithms need more data points than parameters to be able to ‘choose’ a solution, because they cannot estimate a given number of parameters with less than or at least an equal number of data points. It is likely to have similar types of data for many individual cultures, resulting in ‘replicates’ of the same data structure without increasing the numerical rank. Working with data coming from a high number of independent runs, therefore, does not add to the information content and it does not increase the numerical rank. Hence, the analysis is still restricted with respect to the availability of different methods and tools for analysis. However, having replicates could help in decreasing the noise at time points for which information is already available [52].

Another important challenge regarding the processing of the BPD data stems from the unit and the scaling of the parameter values. There are large differences in magnitude of the measurements recorded for different parameters; for instance, pH measurement and the viable cell density can easily vary by six orders of magnitude, making the dataset unsuitable for a number of applications such as Principal Component Analysis or PLSR. A simple solution to overcome this problem is data normalisation, which is the scaling of the numerical values to confine within similar or comparable magnitudes while conserving the trends across time for each parameter. Earlier analyses showed that scaling does not change the inherent nature of the data or the biological interpretation of the results [52].

Standard BPD data stored in the repositories contain dependent or strongly correlated parameters such as viable cell density and viability. Since any parameter reported by an analytical instrument is automatically stored, the identification of dependencies and strong correlations presents yet another challenge that needs to be addressed prior to analysis.

Parameters that are monitored throughout a biopharmaceutical production process may evolve over time, displaying a time-dependent profile, such as total cell density or viable cell density do. Other parameters either remain within a tolerable limit without displaying any particular trends such as cell compactness or the diameter of the cells, or are controlled to be maintained within an allowable limit, such as the pH of the culture. Due to this heterogeneity in time-dependent responses, it is important to ensure that a combination of methods is employed in data pre-processing, which is able to address these differences and capture the relevant trends. This can become particularly challenging in addressing the problem of missing data. Employing a combination of methods that can capture the complex trends in different parameters and how they evolve over time can be helpful in gap filling with introducing only minimal bias.

The format in which online parameters were recorded in the repositories would impose further challenges in data pre-processing. Online parameters such as pH can be recorded with sub-second precision. In contrast, the offline parameter reading at the time of sampling is recorded as the representative value for the day or the hour, depending on the growth rate of the system employed for production. The selection of the numerical values for the online parameters, which would complement the offline parameters at those time points, then becomes a challenge since the most suitable representative should be selected amongst a large pool of consecutive and frequent measurements recorded during a period of a whole hour or a full day. Algorithms that can extract meta-features could facilitate this selection process.

## Outlook

**Fig. 2:**
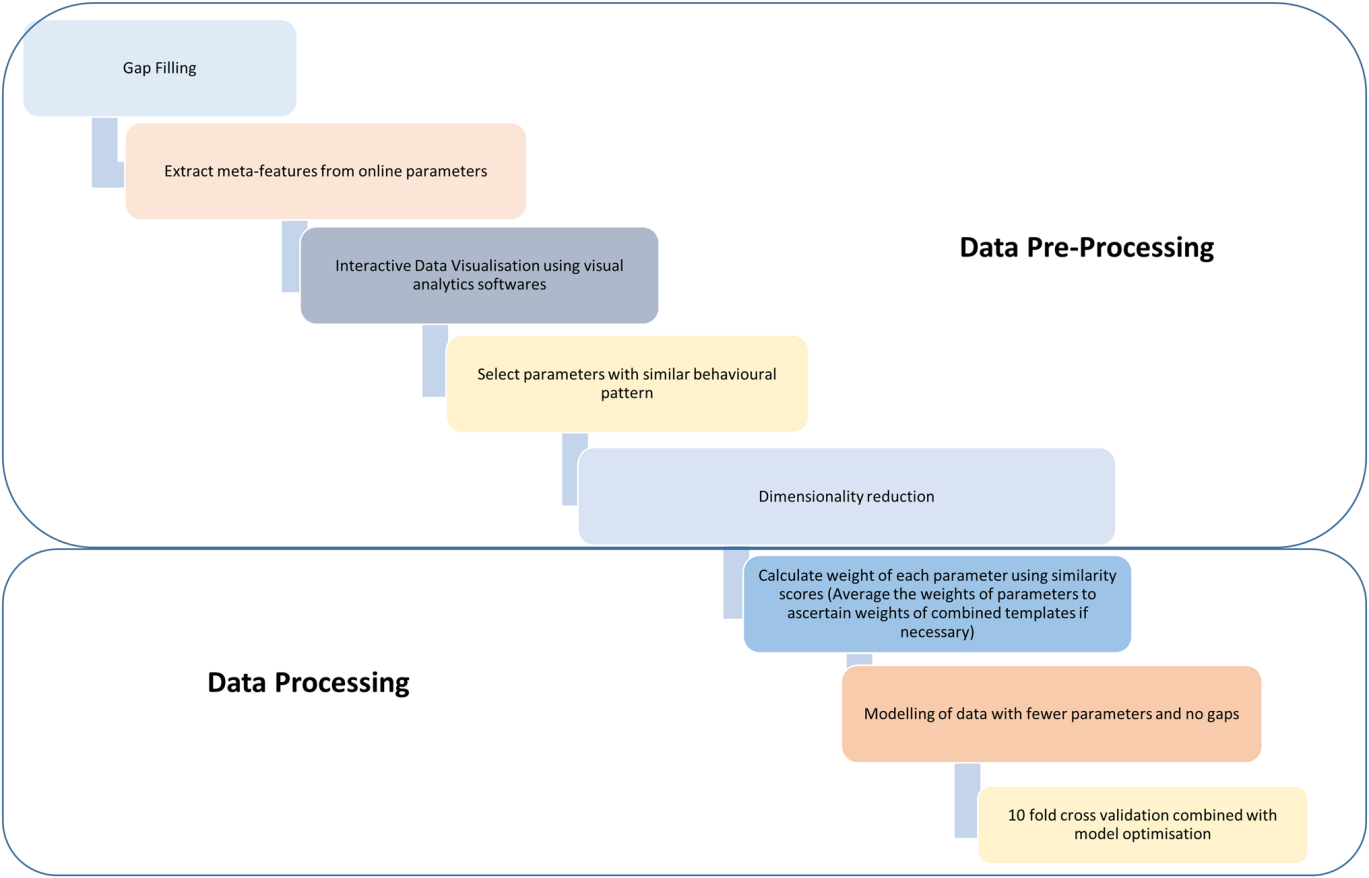
Proposed methodology for biopharmaceutical process development data analysis. Flow diagram with boxes representing sequence of events in data analysis, which can be broadly classified into data pre-processing and data processing

In light of the specific challenges that need to be addressed in the mining and analysis of BPD datasets, an outline summary is proposed for the retrospective analysis of historical BPD data (Fig. 2). The analysis, almost without exceptions, would necessitate the pre-processing of the data. In light of the complex nature of the datasets elaborated above, the selection of a hybrid strategy for the imputation of missing values in the datasets would be encouraged strongly to address the problem of gap filling. The extraction of meta-features from online datasets would be recommended over the use of simpler approaches. Selecting parameters with similar behavioural patterns would be important for dimensionality reduction. A suitable interactive data visualisation software can effectively serve this purpose. Shape-based template-matching framework is a promising method for dimensionality reduction in time series datasets. The processing of the data would be facilitated by assigning weights to the parameters to be employed in the model. Following its construction and its validation, the model can further be optimised to improve its predictive accuracy. This outline proposes a strategy to handle many types of BPD data, although the differences in the nature and processing requirements of different datasets should always be noted. Inevitably, this nature will present the ultimate challenge in the selection of suitable methods for analysis.
